# The Prevalence and Association of Motivational Factors to Exercise in Middle-Aged Sedentary Females: A Cross-Sectional Study

**DOI:** 10.7759/cureus.92075

**Published:** 2025-09-11

**Authors:** Khyati Patel, Bhavana R Gadhavi, Geeta D Bhatt, Rupali Shevalkar

**Affiliations:** 1 Department of Physiotherapy, Parul University, Vadodara, IND; 2 Department of Physiotherapy, Parul institute of Physiotherapy, Parul University, Vadodara, IND; 3 Department of Neurophysiotherapy, KJ Somaiya College of Physiotherapy, Mumbai, IND; 4 Department of Physiotherapy, KJ Somaiya College of Physiotherapy, Mumbai, IND

**Keywords:** exercise, extrinsic motivators, intrinsic motivators, middle-aged females, sedentary females

## Abstract

Introduction

Middle age is associated with increased sedentary behavior and heightened risk of chronic diseases. Although health benefits motivate physical activity, middle-aged women often face significant barriers to engaging in it. Thus, following the need to promote exercise in this age group, the study aimed to explore the motivational factors and the association of these factors that influence middle-aged females while undertaking physical activity using the Revised Motivation for Physical Activity Measure for Females (RM4-FM) questionnaire.

Methodology

A total of 123 females aged 30 to 50 years attending the Fitness and Wellness outpatient department (OPD) of a tertiary care center were enrolled in this cross-sectional study per the inclusion and exclusion criteria. The demographic details of the participants were noted along with BMI and comorbidities. Individuals who were unable to comprehend the questionnaire or were already engaged in any form of exercise were excluded from the study. The eligible participants completed a self-administered RM4-FM questionnaire.

Results

The study reported 61.8% overweight females, followed by 25.2% obese females. The majority of the females, i.e., 32.52%, were diagnosed with hypertension and diabetes mellitus together. Around 56.9% of females were intrinsically motivated for exercise, and 43.1% were extrinsically motivated to exercise as determined by the relative autonomy index (RAI) of the RM4-FM questionnaire. A Pearson’s chi-square test of independence reported a statistically insignificant association between comorbidities and factors, as well as between BMI and factors.

Conclusion

The majority of the females understood the importance of exercise for health benefits clinically, but lacked motivation to comply with physical activity and indulge in an exercise program. Also, the statistically insignificant associations determined that females lack the influence of comorbidities and BMI to adhere to physical activity.

## Introduction

Middle age represents a critical transitional period characterized by increasing personal and professional responsibilities, often leading to heightened physical and mental stress, which is often associated with reduced physical activity and a sedentary lifestyle [1]. Sedentary behaviors reduce muscle glucose uptake, lipoprotein lipase activity, and protein transporter activities, impair lipid metabolism, and diminish carbohydrate metabolism. Furthermore, it decreases cardiac output and systemic blood flow while activating the sympathetic nervous system, ultimately reducing vascular function and insulin sensitivity [2]. Increased sedentary time leads to adiposity and weight gain due to impaired gravitostat and weight homeostat of the body [3]. Several studies have reported that sedentary behavior escalates with advancing age, contributing significantly to the risk of chronic co-morbidities such as obesity, cardiovascular diseases, and type 2 diabetes [2-5]. Middle-aged individuals are particularly vulnerable to these negative health outcomes compared to younger populations [6,7].

Health and fitness have been consistently identified as important motivators for physical activity across adult populations. However, middle-aged women particularly emphasize the health benefits of exercise more than their male counterparts [8-10]. This greater prioritization is often linked to heightened awareness of health risks associated with inactivity, including metabolic disorders and functional decline, along with hormonal dissociation due to closing the timeline for menopause [11,12]. Nevertheless, multiple barriers hinder sustained physical activity among middle-aged women, including time constraints, caregiving duties, lack of social support, and physical discomfort [13]. Thus, considering the essential role of physical activity in preventing lifestyle-related diseases, the primary objective of the present study was to assess the prevalence of the factors that motivate physical activities in middle-aged females. Also, the secondary objective was to study the association of factors that influence middle-aged women to engage in regular physical activity using the Revised Motivation for Physical Activity Measure for Females (RM4-FM) questionnaire (see Appendix A) [14].

## Materials and methods

Study design

This is a cross-sectional study, which is a part of a large mixed-method research project, aimed to evaluate the knowledge and effect of the exercise buddy system training approved by the Institutional Ethics Committee of KJ Somaiya Medical College and Hospital (Mumbai, MH, IND) (approval no. IEC-Feb2023-06). The study was conducted among 123 females aged 30 to 50 years, who attended the Fitness and Wellness outpatient department (OPD) of a tertiary care center after obtaining written informed consent. The inclusion criteria included females within the specified age range who consented to participate and were not currently engaged in any form of structured exercise program. Individuals who were unable to comprehend the questionnaire due to language barriers, had cognitive difficulties, or other impairments, as well as those already participating in regular physical exercise, were excluded from the study.

Sampling and data collection

The sample size was calculated based on the average footfall in the fitness and wellness OPD, following an OPD availability and recruitment window of 25 to 27 eligible middle-aged women, recruiting 150 to 162 women for a duration of six months. Further, a conservative consent response rate of 80% was assumed, yielding a maximum of 130 women. Furthermore, a dropout rate of 5% was observed (seven women), providing a sample size of 123 women. Demographic and clinical information, including BMI and relevant comorbidities, was recorded on a structured data collection form. The eligible participants were asked to complete the RM4-FM questionnaire, a validated self-administered instrument designed to assess motivation for exercise across various dimensions [14]. The questionnaire assesses both intrinsic and extrinsic motivational factors related to physical activity participation.

The RM4-FM comprises four dimensions, namely external regulation, introjected regulation, identifiable regulation, and internal motivation. Each dimension's score is determined by taking the mean item scores in that dimension, and stronger motivational influence within the particular dimension is indicated by higher scores. Additionally, the scores of all motivation dimensions are included to construct the relative autonomy index (RAI): RAI=2×intrinsic motivation+identified regulation−introjected regulation−2×external motivation. The proportional influence of intrinsic and extrinsic elements on physical activity motivation is determined using RAI. A positive RAI value suggests that intrinsic elements have a greater influence on motivation and, consequently, autonomy, whereas a negative RAI value suggests that extrinsic factors have a stronger influence on motivation.

Statistical analysis

Data were analyzed using SPSS Statistics version 25.0 (IBM Corp., Armonk, NY, USA). Continuous variables such as age and BMI were presented as mean ± standard deviation, and categorical variables, including motivation type, obesity status, and comorbidities, were expressed as frequencies and percentages. The qualitative data of association had been analyzed using Pearson's chi-square test, and a p-value of >0.05 was considered statistically significant.

## Results

A total of 123 middle-aged females participated in the study, with a mean age of 46.06±5 years and a mean BMI of 28.38±3.46 kg/m². A majority of the participants were classified as overweight (61.8%), followed by obese (25.2%). The most common comorbidity among participants was the coexistence of hypertension and diabetes mellitus, observed in 32.52% of the study population. Regarding exercise motivation assessed through the RM4-FM questionnaire following RAI, 56.91% of participants were intrinsically motivated, while 43.09% were extrinsically motivated. A Pearson’s chi-square test was used to estimate the association between comorbidities of participants and extrinsic and intrinsic motivational factors, as shown in Table 1. The association between BMI and extrinsic and intrinsic motivational factors was also determined using the same test and is demonstrated in Table 2.

**Table 1:** Association between comorbidities of participants and extrinsic and intrinsic motivational factors

Comorbidities	Extrinsic factors	Intrinsic factors	All	Pearson’s chi-square	p-value
Coronary artery disease	2	4	6	5.335	0.502
Diabetes mellitus	3	6	9		
Diabetes mellitus and hypertension	15	25	40		
Hypertension	7	11	18		
Hypothyroid	16	10	26		
None	5	9	14		
Polycystic ovarian disease	5	5	10		
All	53	70	123		

**Table 2:** Association between BMI and extrinsic and intrinsic motivational factors

BMI	Extrinsic factors	Intrinsic factors	All	Pearson’s chi-square	p-value
Normal weight	5	11	16	1.204	0.548
Overweight	35	41	76		
Obese	13	18	31		
All	53	70	123		

## Discussion

This cross-sectional exploratory study aimed to assess the motivational factors and to study the association with extrinsic and intrinsic factors influencing exercise participation among middle-aged females. The findings revealed that intrinsic motivation was the dominant factor driving exercise behavior, especially among women with existing chronic illnesses such as hypertension and diabetes mellitus. Katanolli et al. [15] mentioned that high rates and poor control of diabetes and hypertension were observed in a cohort of participants aged 40 years or older, emphasizing the urgent need for targeted lifestyle interventions in this age group. Also, a high prevalence of obesity was observed in the current study, differentiating the association and the need for lifestyle improvement. Participants with chronic comorbidities such as hypertension and diabetes demonstrated significantly higher intrinsic motivation compared to those without chronic conditions.

The high rates of obesity and associated comorbidities in this cross-sectional study are consistent with previous studies conducted by Sengupta et al. [5] and Goyal et al. [3] in India, which have reported a sharp increase in lifestyle-related disorders among middle-aged women due to urbanization, sedentary behavior, and hormonal changes associated with aging, mentioning the need for improvement in their quality of life. These findings specifiy the importance of focusing on preventive health measures during midlife to reduce the long-term burden of non-communicable diseases.

Exercise motivation plays a critical role in promoting adherence to physical activity [16,17]. In the present study, the intrinsic factors, such as health benefits and weight management, were stronger motivators than extrinsic factors like social approval or external rewards. This pattern is supported by Geller et al. [18], who found that those who successfully maintained regular physical activity reported higher intrinsic and extrinsic motives with internalized health concerns, which were more consistent in maintaining exercise habits over time. Similarly, Bucciarelli et al. [11] noted that awareness of health risks such as cardiovascular disease and diabetes significantly influenced women’s decisions to engage in physical activity.

The contributing factors for the women being extrinsically motivated are external regulation and introjected regulation, and for being intrinsically motivated are the categories of intrinsic motivation and identified regulation. The present study showed that the categories about intrinsic motivation have higher overall scores than extrinsic motivators, which was consistent with the findings of Teixeira et al. [17] who reported that intrinsic motivation was more predictive for long-term exercise adherence.

Interestingly, although employment status might influence time availability, the present study found no significant association between employment and exercise motivation. This suggests that health-driven intrinsic motivation transcends occupational barriers, which is consistent with the findings reported by Limbers et al. [19], who observed that both working and non-working women prioritize health concerns when motivated to exercise.

A statistically insignificant association between comorbidities and intrinsic motivation is reported in the current study, which highlights an important opportunity for public health interventions. Similarly, Booth et al. [20] also suggested that women diagnosed with chronic co-morbidities remained least receptive to exercise counseling and structured wellness programs that emphasize personal health ignorance. Therefore, integrating motivational strategies for physical activities into chronic comorbidity management programs could enhance patient engagement and outcomes [21].

Limitations

Considering the type of study as cross-sectional, it lacks establishing a correlation between the comorbidities and higher intrinsic motivation. Additionally, the participants were enrolled in a study at a single tertiary care center, limiting the generalizability of the findings to the broader population. The reliance on self-reported data may also introduce recall and reporting biases. Future research should focus on multi-center, longitudinal studies to explore direct relationships and to evaluate the effectiveness of tailored intervention programs in promoting sustained exercise habits among middle-aged women.

## Conclusions

This study highlights that intrinsic motivation, particularly driven by health benefits and weight management, is the primary factor encouraging middle-aged females to engage in physical activity. The presence of chronic illnesses such as hypertension and diabetes mellitus was significantly associated with higher intrinsic motivation. These findings highlight the need for targeted interventions that emphasize the health-related advantages of exercise to promote sustained physical activity among middle-aged women. Future programs should focus on strengthening intrinsic motivators to improve long-term adherence to healthy lifestyles in this population.

## Figures and Tables

**Figure 1:**
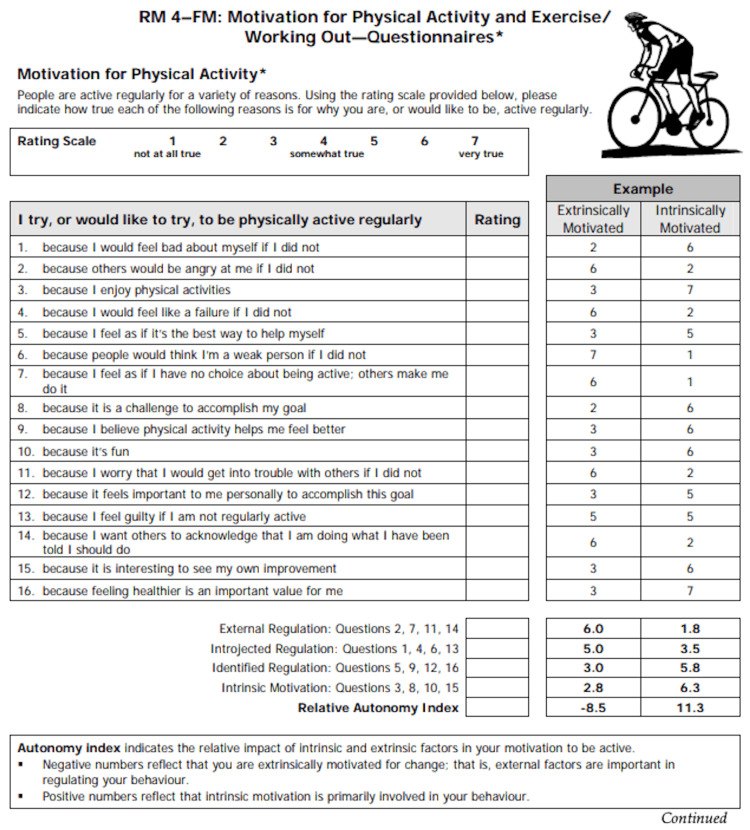
Page one of the RM4-FM questionnaire page 1 The questionnaire is available in the public domain for free use under the terms of the Creative Commons Attribution Non-Commercial License, which permits unrestricted non-commercial use, distribution, and reproduction in any medium, provided the original work is properly cited. From *Validity and reliability of the motivation for physical activity (RM4-FM) questionnaire*. Uimonen M, Repo JP, Grönroos K, Häkkinen A, Walker S, 2021, Journal of Exercise Rehabilitation; 17 (2): 103-111 [14]

**Figure 2:**
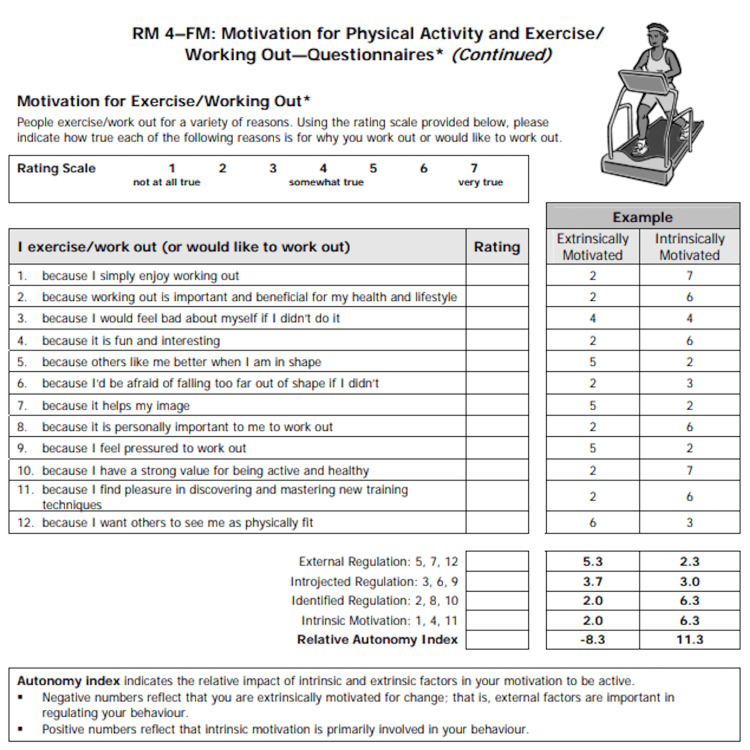
Page two of the RM4-FM questionnaire page 1

## Appendices

Appendix A

Figures 1-2 feature the RM4-FM questionnaire utilized in this study.
